# Validity of Cardiovascular Disease Event Ascertainment Using Linkage to UK Hospital Records

**DOI:** 10.1097/EDE.0000000000000688

**Published:** 2017-05-01

**Authors:** Mika Kivimäki, G. David Batty, Archana Singh-Manoux, Annie Britton, Eric J. Brunner, Martin J. Shipley

**Affiliations:** From the a Department of Epidemiology and Public Health, University College London, London, United Kingdom; b Clinicum, Faculty of Medicine, University of Helsinki, Helsinki, Finland; and c INSERM U1018, Université Paris-Saclay, Villejuif, France.

## Abstract

**Background:**

Use of electronic health records for ascertainment of disease outcomes in large population-based studies holds much promise due to low costs, diminished study participant burden, and reduced selection bias. However, the validity of cardiovascular disease endpoints derived from electronic records is unclear.

**Methods:**

Participants were 7860 study members of the UK Whitehall II cohort study. We compared cardiovascular disease ascertainment using linkage to the National Health Service’s Hospital Episode Statistics database records (hereafter, “HES-ascertainment”) against repeated biomedical examinations—our gold standard ascertainment method (Whitehall-ascertainment). Follow-up for both methods was from 1997 to 2013 for coronary heart disease and from 1997 to 2009 for stroke.

**Results:**

We identified 950 prevalent or incident nonfatal coronary heart disease cases and 118 prevalent or incident nonfatal stroke cases using Whitehall-ascertainment. The corresponding figures for HES-ascertainment were 926 and 107. For coronary heart disease, the sensitivity of HES-ascertainment was 70%, positive predictive value 72%, specificity 96%, and the negative predictive value 96%. The pattern of results for stroke was similar. These statistics did not differ in analyses stratified by age, sex, baseline risk factor status, or after exclusion of prevalent cases. Estimates of risk factor–disease associations were similar between the two ascertainment methods. Including fatal cardiovascular disease in the outcomes improved the agreement between the methods.

**Conclusion:**

Our analyses support the validity of cardiovascular disease ascertainment using linkage to the UK Hospital Episode Statistics database records by showing agreement with high resolution disease data collected in the Whitehall II cohort.

## Abstract 2

Supplemental Digital Content is available in the text.

Accurate ascertainment of disease endpoints is a key tenet of epidemiology. In well-characterized cohort studies of disease etiology, such as Framingham (United States) and the Whitehall II study (United Kingdom), disease outcomes are ascertained using serial biomedical evaluations of the participants.^1–3^ In contrast to this resource-intensive method, current “big data” approaches capitalize on linkage to routinely collected electronic health records to identify incident and prevalent disease.^4^ Data linkage holds much promise due to lower costs, reduced study participant burden, and diminished selection bias.^5,6^ However, the validity of routinely collected records is unclear.

In this study, we used repeated biomedical examinations from the Whitehall II study as the gold standard for ascertainment of cardiovascular disease (hereafter, “Whitehall-ascertainment”),^7,8^ comparing this against disease ascertainment using linkage of study members to the UK National Health Service’s Hospital Episode Statistics (HES) database records (hereafter, “HES-ascertainment”).

## METHODS

### Study Population

The source population of the British Whitehall II study was all London-based, nonindustrial government workers, 35–55 years old, working in 20 departments at study baseline in 1985–1988. With a response of 73%, the baseline cohort consisted of 10,308 employees (6895 men and 3413 women). Ethical approval for the Whitehall II study, including linkage to HES and mortality records, was obtained from University College London Medical School committee on the ethics of human research (reference number 85/0938) and the London-Harrow and Scotland A Research Ethics Committees on the Ethics of Human Research. All participants provided written informed consent.

The health care system in the United Kingdom, National Health Service (NHS), is funded from taxation to provide comprehensive health care coverage available to all individuals legally registered as residents in the United Kingdom. All UK citizens have a unique NHS identification number. The HES is an administrative NHS database containing details of all admissions, outpatient appointments, and accident and emergency attendances at NHS hospitals. Hospitals are paid for the care they deliver based on clinical information from HES about diagnoses and procedures. HES data are also used for healthcare planning, commissioning services, development of national policy, and research (http://content.digital.nhs.uk/hes). The Office of National Statistics, the recognized national statistical institute in the United Kingdom, maintains vital events data, including records of deaths occurring anywhere in the United Kingdom, and for research purposes, these records are distributed by NHS Digital.

### Design

Both Whitehall- and HES-ascertained events were available from the third (clinic 3) to the sixth clinical examination (clinic 6) for coronary heart disease and from clinics 3 to 5 for stroke. Clinic 3 (1997–1999) represents the baseline for the present study, with subsequent examinations taking place in 2003–2004 (clinic 4), 2008–2009 (clinic 5), and 2012–2013 (clinic 6).

### Baseline Characteristics

Demographic characteristics (age, gender, three-level socioeconomic status), smoking (current, ex-, never smoker), hypertension (systolic blood pressure ≥140 mmHg, diastolic blood pressure ≥90 mmHg, or on antihypertensive medication), and high cholesterol (total cholesterol ≥6 mmol/L or on lipid-lowering medication) were measured using standard protocols.^3^

### Ascertainment of Coronary Heart Disease

Whitehall-ascertained nonfatal coronary heart disease was based on 12-lead resting electrocardiogram (ECG) recording, coded using the Minnesota system, and on self-reported coronary heart disease that had been corroborated with information from the general practitioner or by manual retrieval of hospital records. The ascertainment included nonfatal myocardial infarction, definite angina, reported coronary artery bypass grafting, and percutaneous transluminal coronary angioplasty.^7^

HES-ascertainment was based on data linkage to records from hospitalizations for nonfatal coronary heart disease as a primary or secondary diagnosis (defined using the International Statistical Classification of Diseases and Related Health Problems, version 9 [ICD-9] codes 410–414, ICD-10 codes I20–I25, or procedures K40–K49, K50, K75, U19), by using the NHS identification number.

The main outcome was the first incident or recurrent nonfatal coronary heart disease event after baseline. To capture both nonfatal and fatal coronary heart disease in a subsidiary analysis, records of coronary death (defined using ICD-9 codes 410–414 and ICD-10 codes I20–I25) were added to both ascertainment methods. Death records were obtained from data linkage to the Office of National Statistics death registry by using the NHS identification number, and the data included death date and the underlying cause.

### Ascertainment of Stroke

Whitehall-ascertainment for nonfatal stroke was based on self-reported diagnosis and use of the WHO Multinational MONItoring of trends and determinants in CArdiovascular disease (MONICA)-Augsburg stroke questionnaires that capture symptoms associated with events, even if the participant did not report having had a diagnosis. If a participant responded positively to at least one of these, their histories were corroborated with the general practitioner’s confirmation, HES data linkage (ICD codes in HES-ascertainment), or manual retrieval of hospital medical records reviewed by a stroke clinician.^8^

HES-ascertainment was based on data linkage to electronic records from hospitalizations due to stroke as a primary or secondary diagnosis (defined using ICD-9 codes 430, 431, 434, 436 and ICD-10-cased I60, I61, I63, I64).

The first incident or recurrent nonfatal stroke after baseline was the main outcome. As in relation to coronary heart disease, fatal or nonfatal stroke was an additional outcome; records from data linkage to the Office of National Statistics death registries (the same ICD codes) were added to both ascertainment methods.

### Statistical Methods

To examine the validity of HES-ascertained coronary heart disease and stroke using Whitehall-ascertainment as the gold standard, we computed the sensitivity (the proportion of Whitehall-ascertained cases that are detected with HES-ascertainment), specificity (the proportion of participants without Whitehall-ascertained disease who have no HES-ascertained disease), positive predictive value (the proportion of participants with HES-ascertained disease that are Whitehall-ascertained cases), and negative predictive value (the proportion of participants without HES-ascertained disease that are Whitehall-ascertained noncases). These statistics with 95% confidence intervals were computed separately for incident/recurrent nonfatal events and fatal/nonfatal events as the outcome. The results were reported for the total cohort and by age group (<55, 55–59, ≥60 years), sex, socioeconomic status, smoking, hypertension, hypercholesterolemia and period of follow-up (from clinics 3 to 4, from clinics 4 to 5, and from clinics 5 to 6), and after excluding prevalent cases of coronary heart disease and stroke at baseline. We also computed age- and sex-adjusted associations of risk factors (age, sex, socioeconomic status, smoking, high blood pressure, and high cholesterol) with coronary heart disease and stroke using the two methods of disease ascertainment.

## RESULTS

A total of 7855 study members (76.2% of the 10,308 initial study members) participated in clinic 3 and had follow-up for coronary heart disease based on both the Whitehall- and the HES-ascertainment. The corresponding number for the stroke analysis was 7,860. Mean age of the participants was 56 years at baseline, and 30% were women. A flowchart for sample selection is provided in eAppendix 1; http://links.lww.com/EDE/B213.

During surveillance, we identified 950 incident or recurrent nonfatal coronary heart disease cases and 118 incident or recurrent nonfatal stroke cases using Whitehall-ascertainment methods. The corresponding figures for HES-ascertainment were similar but slightly lower (926 and 107). In Table 1, we show that using Whitehall-ascertainment as the referent, the sensitivity of HES-ascertainment for coronary heart disease was 70% and the positive predictive value was 72%. These statistics were somewhat higher for men (72% and 75%) than for women (61% and 59%). Specificity and negative predictive values varied between 93% and 98% in the total cohort and in age and sex groups. Exclusion of participants with prevalent disease had little impact on these results. In Table 2, we see that the pattern of results for stroke was similar. Specificity and negative predictive values were 99% or higher in all cases.

**TABLE 1.**
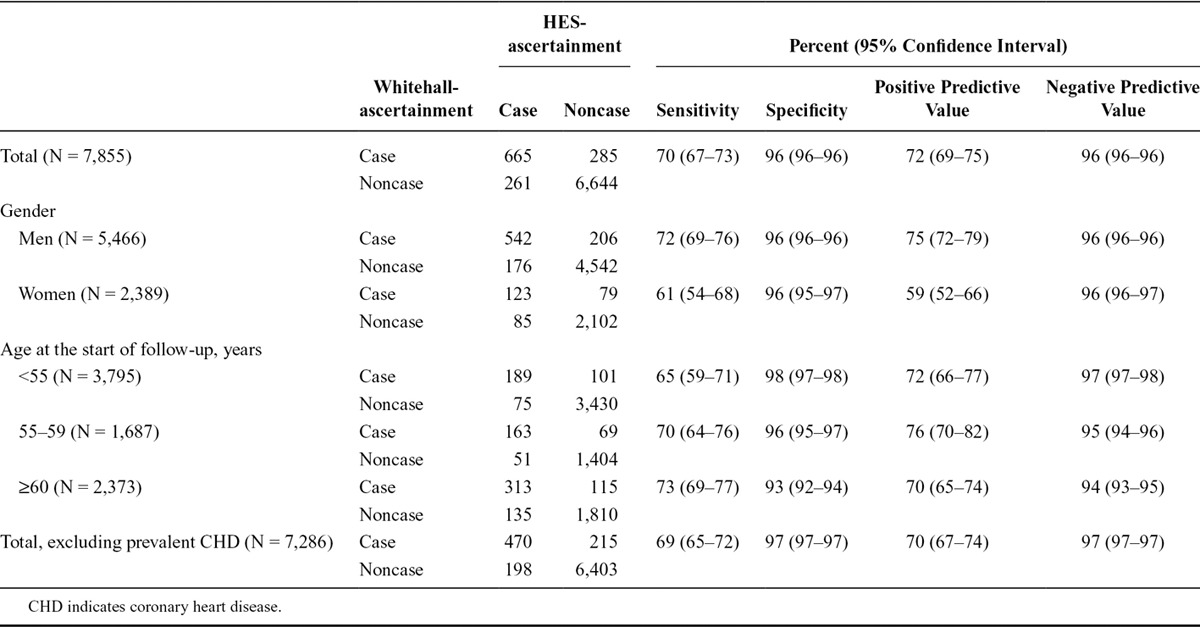
Cross Classification and Validation of Nonfatal Incident or Recurrent CHD Defined Using HES-ascertainment with Whitehall-ascertainment as the Reference in the Total Cohort and According to Subgroups

**TABLE 2.**
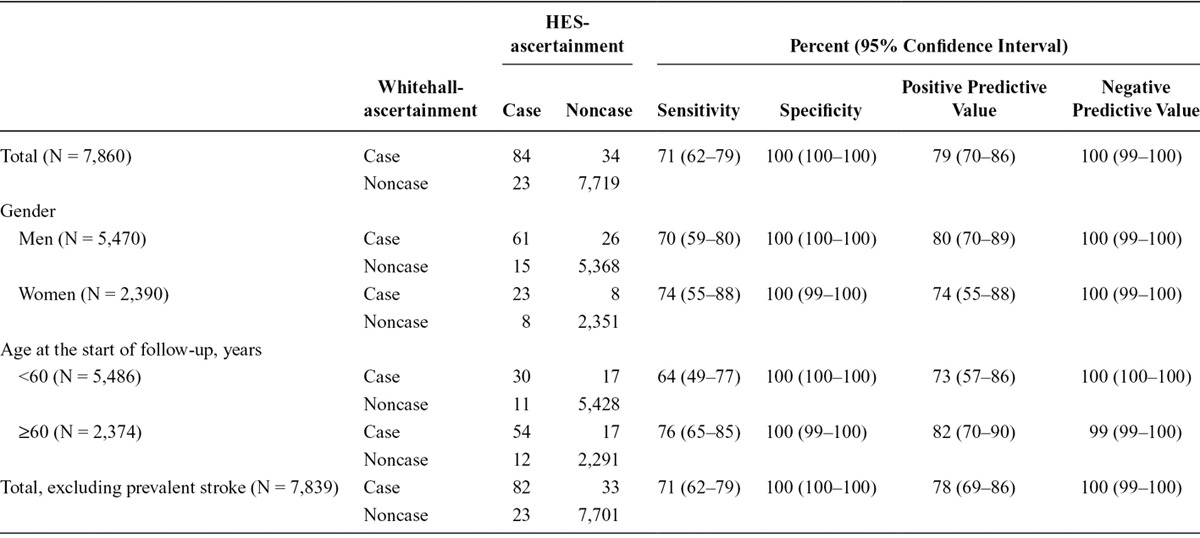
Cross Classification and Validation of Nonfatal Incident or Recurrent Stroke Defined Using HES-ascertainment with Whitehall-ascertainment as the Reference in the Total Cohort and According to Subgroups

In analyses for nonfatal incident or recurrent coronary heart disease stratified by risk factor status, with one exception, sensitivity exceeded 65% and the positive predictive value exceeded 70% (eAppendix 2; http://links.lww.com/EDE/B213). Specificity and negative predictive values varied between 93% and 98%. Sensitivity improved over time (eAppendix 3; http://links.lww.com/EDE/B213): for coronary heart disease, it was 52% between clinics 3 and 4, but 78% between clinics 5 and 6. For stroke, sensitivity was 64% in the first period and 75% between subsequent clinics 4 and 5. Irrespective of the period of follow-up, specificity and negative predictive values were high (≥96%).

The associations of risk factors with coronary heart disease and stroke did not differ between Whitehall- and HES-ascertained endpoints (eAppendices 4 and 5; http://links.lww.com/EDE/B213). Supplementary analyses on the comparison of Whitehall- and HES-ascertainment for nonfatal or fatal cardiovascular disease as the outcome are provided in eAppendices 6–9; http://links.lww.com/EDE/B213. For Whitehall-ascertainment, death records identified 69 new coronary heart disease cases (total N for cases = 1,019) and six new stroke cases (total N = 124). The corresponding figures for HES-ascertainment were 72 (total N = 998) and 15 (total N = 122). The agreement between the two methods improved slightly.

## DISCUSSION

Our analyses support the validity of cardiovascular disease ascertainment using linkage to HES, the UK’s nationwide hospital events database, by showing good agreement with high resolution data collected in the Whitehall II cohort. The estimates of associations between classic risk factor and cardiovascular diseases were also very similar for each of the two ascertainment methods, as would be expected given the high specificity and apparently nondifferential sensitivity.^9^

In validation studies of electronic records, the reference standard has varied, including, e.g., general practitioner (physician)-verified events, patient self-report based on interviews, independent clinical registries, laboratory information system databases, pathology registries, biobanks, and autopsy reports.^10–12^ We used serial biomedical evaluations combined with clinical data tracing as the gold standard in a context of an unusually well-characterized cohort study. This comparison of the traditional resource-intensive ascertainment method used in longitudinal cohort studies^1,2^ with the low-cost alternative data linkage method indicates that, at least in the United Kingdom, linkage with electronic health records is suitable for detecting major cardiovascular disease events for many epidemiologic purposes.

Thirty percent of the Whitehall-ascertained incident and recurrent nonfatal coronary heart disease cases were not identified by HES-ascertainment. The corresponding percentage for stroke was 29%. Although some of these cases are likely to be due to the limited coverage of HES data, especially in the early years of the follow-up, some of the uncaptured cases also included angina events that did not result in hospitalization.^13^

A total of 28% of the coronary heart disease and 21% of stroke cases that were captured by HES were not captured by Whitehall-ascertainment. These cases are likely to be true cases rather than errors in HES database. Whitehall-ascertainment may miss cases if the participant does not attend a clinical examination or does not respond to questionnaires that trigger additional corroboration against general practitioner notes and manual retrieval of medical records from hospitals. A further limitation of Whitehall stroke ascertainment was the absence of brain scanning.

The electronic health records are integral to the new precision medicine in cardiology^6^ and studies evaluating such databases for large-scale research support their utility.^10–12^ In the UK Biobank, e.g., linkage of over 330,000 study members to records from HES has been shown to be both a pragmatic method to identify cardiovascular disease and one that minimizes participant burden.^5^ Our findings suggest that use of UK HES records is a valid method for coronary heart disease and stroke ascertainment for cohort studies examining risk factor–disease associations. It offers a low-cost alternative to traditional ascertainment through biomedical screening and tracing processes.

## ACKNOWLEDGMENTS

We thank all of the participating civil service departments and their welfare, personnel, and establishment officers; the British Occupational Health and Safety Agency; the British Council of Civil Service Unions; all participating civil servants in the Whitehall II study; and all members of the Whitehall II study team. The Whitehall II study team comprises research scientists, statisticians, study coordinators, nurses, data managers, administrative assistants, and data entry staff, who make the study possible.

## Supplementary Material
