# Supplementary material for: Validity of Cardiovascular Disease Event Ascertainment Using Linkage to UK Hospital Records
Source: Epidemiology. 2017 May 1;28(5):735–9. doi: 10.1097/EDE.0000000000000688 (PMC5540351; doi:10.1097/EDE.0000000000000688)
Supplement: Supplementary file 1 [file ede-28-735-s001.pdf]

eAppendices

eAppendix 1. Flow Chart of Sample Selection

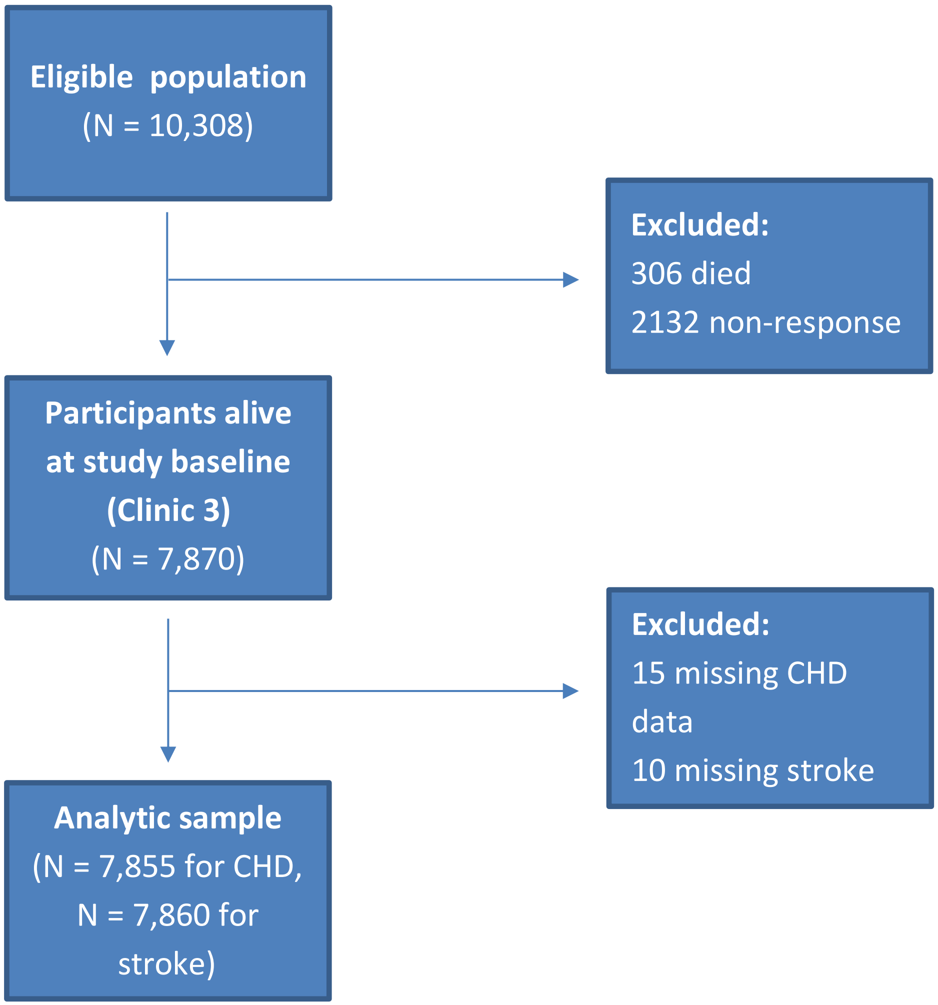

eAppendix 2. Cross-classification and Validation of Non-fatal Incident or Recurrent Coronary Heart Disease Defined Using HES-ascertainment with Whitehall-ascertainment as the Reference According to Risk Factor Sub-groups

Risk factor		Whitehall-ascertainment	HES-ascertainment		Percent (95% confidence interval)			
			Case	Non-case	Sensitivity	Specificity	Positive predictive value	Negative predictive value
Socioeconomic status	High (N=3246)	Case	260	122	68 (63-73)	97 (97-98)	76 (71-81)	96 (95-97)
		Non-case	82	2782				
	Intermediate (N=3429)	Case	315	125	72 (67-76)	96 (95-97)	72 (68-77)	96 (95-97)
		Non-case	120	2869				
	Low (N=1180)	Case	90	38	70 (62-78)	94 (93-96)	60 (52-68)	96 (95-97)
		Non-case	59	993				
Smoking habit	Never (N=3522)	Case	264	117	69 (64-74)	97 (96-97)	71 (67-76)	96 (96-97)
		Non-case	106	3035				
	Former (N=2901)	Case	274	118	70 (65-74)	97 (96-97)	77 (72-81)	95 (95-96)
		Non-case	83	2426				
	Current (N=765)	Case	77	29	73 (63-81)	96 (94-97)	74 (65-82)	96 (94-97)
		Non-case	27	632				
Hypertensive status	Non-hypertensive (N=4584)	Case	285	140	67 (62-72)	98 (97-98)	74 (70-79)	97 (96-97)
		Non-case	98	4061				
	Hypertensive (N=1875)	Case	260	103	72 (67-76)	95 (93-96)	76 (71-81)	93 (92-95)
		Non-case	81	1431				
Cholesterol level	<6.0 mmol/L (N=3315)	Case	207	115	64 (59-70)	98 (97-98)	75 (69-80)	96 (96-97)
		Non-case	70	2923				
	≥6.0 mmol/L or lipid lowering drugs (N=3083)	Case	335	124	73 (69-77)	96 (95-97)	76 (72-80)	95 (94-96)
		Non-case	106	2518				

eAppendix 3. Cross-classification and Validation of Non-fatal Incident or Recurrent Coronary Heart Disease and Stroke Defined Using HES-ascertainment with Whitehall-ascertainment as the Reference According to Period of Follow-up

Outcome	Period of follow for ascertainment of outcome[a]	Whitehall-ascertainment	HES-ascertainment		Percent (95% confidence interval)			
			Case	Non-case	Sensitivity	Specificity	Positive predictive value	Negative predictive value
CHD								
	Clinic 3 to Clinic 4 (N=7855)	Case	214	196	52 (47-57)	99 (98-99)	77 (72-82)	97 (97-98)
		Non-case	63	7382				
	Clinic 4 to Clinic 5 (N=7129)	Case	336	222	60 (56-64)	99 (99-99)	84 (80-84)	97 (96-97)
		Non-case	64	6507				
	Clinic 5 to Clinic 6 (N=6590)	Case	263	73	78 (74-83)	96 (95-96)	50 (45-54)	99 (99-99)
		Non-case	268	5986				
Stroke								
	Clinic 3 to Clinic 4 (N=7860)	Case	30	17	64 (49-77)	100 (100-100)	91 (76-98)	100 (100-100)
		Non-case	3	7810				
	Clinic 4 to Clinic 5 (N=7123)	Case	54	18	75 (63-85)	100 (100-100)	79 (68-88)	100 (100-100)
		Non-case	14	7037				

[a] Years in which Whitehall study Clinics were conducted: 1997-1999 (Clinic 3); 2003-2004 (Clinic 4); 2008-2009 (Clinic 5); 2012-2013 (Clinic 6).

eAppendix 4. Associations of Risk Factors with Incident or Recurrent Whitehall II Ascertained or HES Ascertained Non-fatal Coronary Heart Disease.

		Incident or recurrent coronary heart disease until 2013			
		Whitehall II-ascertainment		HES-ascertainment	
Risk factor at 2003-04	N	No. events	HR[a] (95% CI)	No. events	HR[a] (95% CI)
Age (per 10y)	7855	950	2.3 (2.0-2.5)	926	2.4 (2.1-2.7)
Gender					
Male	5466	748	1.0 (Ref)	718	1.0 (Ref)
Female	2389	202	0.6 (0.5-0.7)	208	0.6 (0.5-0.7)
Socioeconomic status					
High	3246	382	1.0 (Ref)	342	1.0 (Ref)
Intermediate	3429	440	1.3 (1.2-1.5)	435	1.4 (1.2-1.6)
Low	1180	128	1.5 (1.2-1.8)	149	1.5 (1.2-1.9)
Anti-hypertensive medication					
No	6701	661	1.0 (Ref)	634	1.0 (Ref)
Yes	1089	280	2.8 (2.4-3.2)	282	2.6 (2.2-3.0)
Systolic blood pressure (per 10mmHg) in those not on anti-hypertensive medication	5610	575	1.1 (1.1-1.2)	518	1.1 (1.0-1.2)
Lipid lowering medication					
No	7519	853	1.0 (Ref)	814	1.0 (Ref)
Yes	271	88	3.0 (2.4-3.8)	102	3.4 (2.8-4.2)
Cholesterol (per 1 mmol) in those not on lipid lowering medication	6196	712	1.1 (1.1-1.2)	649	1.2 (1.1-1.3)
Smoking habit					
Never	3522	381	1.0 (Ref)	370	1.0 (Ref)
Former	2901	392	1.2 (1.0-1.3)	357	1.1 (0.9-1.2)
Current	765	106	1.6 (1.3-1.9)	104	1.4 (1.1-1.8)

[a] Hazard ratios by age and gender are mutually adjusted and all other hazard ratios are adjusted for age and sex.

eAppendix 5. Associations of Risk Factors with Incident or Recurrent Whitehall II Ascertained or HES Ascertained Non-fatal Stroke.

	Incident or recurrent stroke until 2009				
		Whitehall II-ascertainment		HES-ascertainment	
Risk factor at 2003-04	N	No. events	HR[a] (95% CI)	No. events	HR[a] (95% CI)
Age (per 10y)	7860	118	3.1 (2.3-4.4)	107	3.2 (2.2-4.4)
Gender					
Male	5470	87	1.0 (Ref)	76	1.0 (Ref)
Female	2390	31	0.8 (0.5-1.2)	31	0.9 (0.6-1.3)
Socioeconomic status					
High	3248	43	1.0 (Ref)	36	1.0 (Ref)
Intermediate	3431	49	1.4 (1.2-1.6)	46	1.3 (0.9-2.1)
Low	1181	26	1.9 (1.5-2.3)	25	2.0 (1.1-3.6)
Anti-hypertensive medication					
No	6704	89	1.0 (Ref)	78	1.0 (Ref)
Yes	1088	29	1.6 (1.1-2.5)	29	1.7 (1.1-2.7)
Systolic blood pressure (per 10mmHg) in those not on anti-hypertensive medication	5611	78	1.2 (1.1-1.4)	60	1.2 (1.0-1.3)
Lipid lowering medication					
No	7521	111	1.0 (Ref)	98	1.0 (Ref)
Yes	271	7	1.5 (0.7-3.1)	9	1.9 (1.0-3.8)
Cholesterol (per 1 mmol) in those not on lipid lowering medication	6196	95	0.7 (0.6-0.9)	76	0.7 (0.5-0.9)
Smoking habit					
Never	3524	54	1.0 (Ref)	45	1.0 (Ref)
Former	2901	38	0.8 (0.5-1.2)	33	0.8 (0.5-1.3)
Current	764	19	2.0 (1.2-3.4)	18	2.0 (1.2-3.5)

[a] Hazard ratios by age and gender are mutually adjusted and all other hazard ratios are adjusted for age and sex.

eAppendix 6. Cross-classification and Validation of Fatal and Non-fatal Coronary Heart Disease Defined Using HES-ascertainment with Whitehall-ascertainment as the Reference in the Total Cohort and According to Sub-groups

			HES-ascertainment		Percent (95% confidence interval)			
		Whitehall-ascertainment	Case	Non-case	Sensitivity	Specificity	Positive predictive value	Negative predictive value
Total	(N=7851)	Case	744	275	73 (70-76)	96 (96-97)	74 (71-77)	96 (96-96)
		Non-case	254	6578				
Gender	Men (N=5465)	Case	602	199	75 (72-78)	96 (96-97)	78 (75-81)	96 (95-96)
		Non-case	171	4493				
	Women (N=2386)	Case	142	76	65 (58-71)	96 (95-97)	63 (56-63)	96 (96-97)
		Non-case	83	2085				
Age at start of follow-up	<55 years (N=3793)	Case	212	101	68 (62-73)	98 (97-98)	75 (69-80)	97 (97-98)
		Non-case	71	3409				
	55 – 59 years (N=1685)	Case	175	66	73 (67-78)	96 (95-97)	76 (70-81)	95 (94-96)
		Non-case	56	1388				
	≥ 60 years (N=2373)	Case	357	108	77 (73-81)	93 (92-94)	74 (70-78)	94 (93-95)
		Non-case	127	1781				
Total, excluding prevalent CHD	(N=7283)	Case	530	211	72 (68-75)	97 (97-97)	73 (70-76)	97 (96-97)
		Non-case	195	6347				

eAppendix 7. Cross-classification and Validation of Fatal and Non-fatal Stroke Defined Using HES-ascertainment with Whitehall-ascertainment as the Reference in the Total Cohort and According to Sub-groups

			HES-ascertainment		Percent (95% confidence interval)			
		Whitehall-ascertainment	Case	Non-case	Sensitivity	Specificity	Positive predictive value	Negative predictive value
Total	(N=7856)	Case	91	33	73 (65-78)	100 (99-100)	75 (66-82)	100 (99-100)
		Non-case	31	7701				
Gender	Men (N=5469)	Case	63	25	72 (61-71)	100 (99-100)	79 (69-87)	100 (99-100)
		Non-case	17	5364				
	Women (N=2387)	Case	28	8	78 (61-90)	99 (99-100)	67 (50-80)	100 (99-100)
		Non-case	14	2337				
Age at start of follow-up	<60 years (N=5482)	Case	30	17	64 (49-77)	100(100-100)	65 (50-79)	100 (100-100)
		Non-case	16	5419				
	≥ 60 years (N=2374)	Case	61	16	79 (68-88)	99 (99-100)	80 (70-89)	99 (99-100)
		Non-case	15	2282				
Total, excluding prevalent stroke	(N=7835)	Case	89	32	74 (65-81)	100 (99-100)	74 (65-82)	100 (99-100)
		Non-case	31	7683				

eAppendix 8. Cross-classification and Validation of Fatal and Non-fatal Coronary Heart Disease Defined Using HES-ascertainment with Whitehall-ascertainment as the Reference According to Risk Factor Sub-groups

Risk factor		Whitehall-ascertainment	HES-ascertainment		Percent (95% confidence interval)			
			Case	Non-case	Sensitivity	Specificity	Positive predictive value	Negative predictive value
Socioeconomic status	High (N=3246)	Case	283	119	70 (66-95)	97 (97-98)	78 (74-82)	96 (95-97)
		Non-case	79	2765				
	Intermediate (N=3425)	Case	349	119	75 (70-78)	96 (95-97)	74 (70-78)	96 (95-97)
		Non-case	120	2837				
	Low (N=1180)	Case	112	37	75 (67-82)	95 (93-96)	67 (59-74)	96 (95-97)
		Non-case	55	976				
Smoking habit	Never (N=3520)	Case	292	113	72 (67-76)	97 (96-97)	73 (69-78)	96 (96-97)
		Non-case	106	3009				
	Former (N=2901)	Case	305	113	73 (68-77)	97 (96-97)	79 (75-83)	96 (95-96)
		Non-case	79	2404				
	Current (N=764)	Case	88	29	75 (66-83)	96 (94-97)	77 (68-84)	96 (94-97)
		Non-case	27	620				
Hypertensive status	Non-hypertensive (N=4582)	Case	303	138	69 (64-73)	98 (97-98)	75 (71-80)	97 (96-97)
		Non-case	99	4042				
	Hypertensive (N=1873)	Case	301	97	76 (71-80)	95 (94-96)	80 (75-84)	94 (92-95)
		Non-case	76	1399				
Cholesterol level	<6.0 mmol/L (N=3313)	Case	227	114	67 (61-72)	98 (97-98)	76 (71-81)	96 (95-97)
		Non-case	70	2902				
	≥6.0 mmol/L or lipid lowering drugs (N=3081)	Case	373	118	76 (72-80)	96 (95-97)	79 (75-82)	95 (95-96)
		Non-case	102	2488				

eAppendix 9. Cross-classification and Validation of Fatal and Non-fatal Coronary Heart Disease and Stroke Defined Using HES-ascertainment with Whitehall-ascertainment as the Reference According to Period of Follow-up

			HES-ascertainment		Percent (95% confidence interval)			
Outcome	Period of follow for ascertainment of outcome[a]	Whitehall-ascertainment	Case	Non-case	Sensitivity	Specificity	Positive predictive value	Negative predictive value
CHD								
	Clinic 3 to Clinic 4 (N=7851)	Case	249	190	57 (52-61)	99 (98-99)	71 (66-76)	97 (97-98)
		Non-case	100	7312				
	Clinic 4 to Clinic 5 (N=7119)	Case	360	218	62 (58-66)	99 (98-99)	81 (77-84)	97 (96-97)
		Non-case	87	6454				
	Clinic 5 to Clinic 6 (N=6583)	Case	301	73	80 (76-84)	96 (95-96)	54 (49-58)	99 (98-99)
		Non-case	260	5949				
Stroke								
	Clinic 3 to Clinic 4 (N=7856)	Case	35	16	69 (54-81)	100 (100-100)	73 (58-85)	100 (100-100)
		Non-case	13	7792				
	Clinic 4 to Clinic 5 (N=7121)	Case	56	18	76 (64-85)	100 (100-100)	74 (62-83)	100 (100-100)
		Non-case	20	7027				

[a] Years in which Whitehall study Clinics were conducted: 1997-1999 (Clinic 3); 2003-2004 (Clinic 4); 2008-2009 (Clinic 5); 2012-2013 (Clinic 6).
